# A Preliminary Experimental Study of Polydimethylsiloxane (PDMS)-To-PDMS Bonding Using Oxygen Plasma Treatment Incorporating Isopropyl Alcohol

**DOI:** 10.3390/polym15041006

**Published:** 2023-02-17

**Authors:** Anthony Tony, Ildiko Badea, Chun Yang, Yuyi Liu, Kemin Wang, Shih-Mo Yang, Wenjun Zhang

**Affiliations:** 1 Department of Mechanical Engineering, University of Saskatchewan, Saskatoon, SK S7N 5A9, Canada; 2 College of Pharmacy and Nutrition, University of Saskatchewan, Saskatoon, SK S7N 5E5, Canada; 3 School of Mechatronics and Automation, Shanghai University, Shanghai 200444, China

**Keywords:** PDMS, microfluidics, oxygen plasma, bonding

## Abstract

Polydimethylsiloxane (PDMS) is a widely used material for soft lithography and microfabrication. PDMS exhibits some promising properties suitable for building microfluidic devices; however, bonding PDMS to PDMS and PDMS to other materials for multilayer structures in microfluidic devices is still challenging due to the hydrophobic nature of the surface of PDMS. This paper presents a simple yet effective method to increase the bonding strength for PDMS-to-PDMS using isopropyl alcohol (IPA). The experiment was carried out to evaluate the bonding strength for both the natural-cured and the heat-cured PDMS layer. The results show the effectiveness of our approach in terms of the improved irreversible bonding strength, up to 3.060 MPa, for the natural-cured PDMS and 1.373 MPa for the heat-cured PDMS, while the best bonding strength with the existing method in literature is 1.9 MPa. The work is preliminary because the underlying mechanism is only speculative and open for future research.

## 1. Introduction

Microfluidic devices have found important applications in biology and medicine, leading to the development of new technology such as lab-on-a-chip (LOC), organ-on-a-chip (OOC) [1,2,3,4,5,6], and soft robotics [7]. These devices are called bio-microfluidic devices. To build a bio-microfluidic device, the key requirement is to construct a closure channel or chamber, which further requires the bonding operation of two pieces of materials.

Polydimethylsiloxane (PDMS) is a silicon-based organic polymer widely used for bio-microfluidic devices because of its excellent biocompatibility, high permeability to gases, and transparency [8,9,10,11,12,13,14,15]. PDMS is an inexpensive material compared to glass and silicon, and further PDMS devices can usually be disposable [15,16,17]. Bonding is an essential step in creating multilayer PDMS bio-microfluidic devices. PDMS can be bonded to different substrates, including the PDMS material itself, using different bonding techniques such as thermal bonding [18,19,20,21,22,23], solvent-assisted bonding [10,24,25,26,27,28,29,30,31,32], adhesive-based bonding [24,31,33,34,35], and surface modification bonding [13,36,37,38,39,40,41,42,43,44,45].

Thermal bonding involves polymer melting, which may create significant distortions, limiting its applications in bioMEMS [23]. Thermal bonding in a low-temperature environment may reduce distortion, but the process takes a long time (up to 1 or 2 days). Adhesive-based bonding is low cost and easy to realize, but the distribution of the glue in a selective area on the bonding surface is difficult, which restricts its use in certain applications. UV-curable adhesives can help the selective distribution of the glue by using a mask, but making a mask is costly and time consuming. Poor distribution of the glue may cause the glue or glue residue to enter the channel, thus clogging the channel. Inadequate use of the glue can alter the original properties of the PDMS device and can even create a toxic environment. The bonding of an uncured PDMS with the other can create a high bonding strength between the PDMS and the other, up to about 671 kPa, as reported in [46]. However, the uncured PDMS has difficulty controlling its shape, and this is not suitable to bio-microfluidic devices. Finally, the adhesive-based bonding of the PDMS with the glass shows a low bonding strength [47]. Solvent-assisted bonding is widely used in medical applications that have strict protocols [48]. Solvents have different biocompatibility properties [49], e.g., organic solvents break down polymer chains at the surface [50], and their selection thus needs extreme care. Oxidizing the PDMS surface with the piranha solution (concentrated sulfuric acid and hydrogen peroxide) can enhance solvent-assisted bonding by producing irreversible covalent bonds. However, the presence of additional solvents during bonding may produce swelling of PDMS, which may further alter the original function of the PDMS device [51,52].

Finally, surface modification bonding is perhaps one of the most prevalent bonding methods, in which plasma treatment, a kind of surface modification, predominates. In plasma treatment, the surface of PDMS is modified from a hydrophobic to a hydrophilic state. It is noted that the hydrophilic state of the surface of PDMS is an essential condition for attaining high-quality bonding [53]. In plasma treatment, gas is ionized in a vacuum chamber to form plasma. There are a variety of gases and their vapors, such as O_2_, N_2_, NH_3_, H_2_O, and CO_2_, which are used in plasma treatment [54,55,56]. Among these various gases, oxygen plasma has been shown to achieve the best bonding strength between PDMS and other materials (including PDMS itself), as described in Table 1 [57,58,59,60,61,62]. It is further remarked that oxygen plasma treatment (1) can change the surface of materials in a physical manner to clean the surface [63], (2) can also change the surface plasticity [64], and (3) can be conducted in a controlled manner [65].

However, there are still several challenges in the bonding of PDMS to materials such as SU-8 [9,31], Poly(methyl methacrylate) (PMMA) [10], parylene [12,61], glass [13,37,62,63], silicon [64,65], metal [66], plastic [30,67,68,69], as well as PDMS itself [59]. This paper is focused on the bonding of PDMS to PDMS. The first challenge is to further increase the bonding strength with reference to the highest bonding strength for PDMS-to-PDMS, 1.9 MPa, currently achieved in literature [62] (see Table 1 as well), given that PDMS has unfavorable surface properties such as low surface energy, chemically inertness, and a hydrophobic surface [70,71]. Surface modification can change the surface wettability of PDMS from a hydrophobic to a hydrophilic surface; however, the recovery of the hydrophobic surface can take between one and several minutes, which creates the second challenge in PDMS bonding for building bio-microfluidic devices, i.e., the difficulty of achieving a sufficient precise alignment for the two bonded parts in a stringent time window. It should be mentioned that surface modification remains to be a promising approach to address the two foregoing challenges, especially surface modification with oxygen plasma [59]. For instance, in the literature [72], an approach was proposed to coat the solvents such as methanol, ethanol, isopropanol (IPA), or deionized water to wet the layers after the plasma treatment to prevent hydrophobic recovery. This approach may increase the time window for the hydrophobic recovery of PDMS, thereby facilitating precise alignment. Unfortunately, these coating materials are not found to increase bonding strength, the details of which will be discussed further in Section 2.

In the study reported in this paper, we proposed a new process for PDMS–PDMS bonding, which has three steps: (1) put a liquid (i.e., IPA) between two PDMS layers, (2) assemble them, and (3) treat the assembly with the oxygen plasma in the chamber. It is noted that our process significantly differs from many existing approaches in literature in terms of the order of steps. Specifically, in our approach, the assembly operation (including the alignment operation), i.e., Step (2), is prior to the surface modification operation, i.e., Step (3), but in the existing approaches, the reverse order for these two is taken. Therefore, the second challenge, as mentioned above, has been overcome. We conducted the experiment for the bonding of PDMS to PDMS with and without the presence of IPA. Results showed that the bonding strength was improved greatly in the presence of IPA compared with the situation without IPA, thereby the first challenge, as mentioned above, has been overcome. We also speculated a mechanism behind this result, while leaving a comprehensive study of this mechanism to the future. It is worth mentioning that another important benefit of our approach is that it is free of distortion, presented after the plasma treatment with the existing approaches.

## 2. Related Work

In [73], the bonding of PDMS to glass was studied. Oxygen plasma activation (a kind of surface modification) was taken on PDMS inside a plasma-treatment chamber. After that, the bonded PDMS and glass were placed in a hydraulic press hot plate at 65 °C for 90 min to complete the bonding [73]. In [74], methanol was added to the surface of PDMS during surface activation. Two pieces of surface-activated PDMS were put together in the plasma chamber, specifically over a hot plate at 85 °C to evaporate the methanol and to complete the entire bonding process. It was found that surface activation with methanol had no effect on bonding strength [74].

Tan et al. [75] studied the effect of an extended oxygen plasma treatment on a bonded PDMS structure, which aims to make the bonded surface more hydrophilic. The whole process has two stages. In the first stage, the surface activation of the PDMS was performed with oxygen plasma, and the bonded PDMS assembly was baked in an oven for 2 h at 150 °C. In the second stage, the bonded PDMS assembly was once again exposed to the oxygen plasma treatment (with 70 W constant power from 100 s–500 s) and subsequently immersed into deionized (DI) water, which was expected to pass through the access holes. Finally, the treated PDMS assembly was placed in a vacuum chamber for 7 days to remove air bubbles. However, they did not study the bonding strength, but instead studied the duration of hydrophobic properties [75]. Some fundamental studies were also conducted in [75], which showed that the vacuum plasma environment can prolong the hydrophilicity of the surface of the PDMS, which may be the cause of the improvement of bonding strength. However, the challenge remains in aligning the top PDMS and bottom PDMS (completed in the first stage).

**Figure 1:**
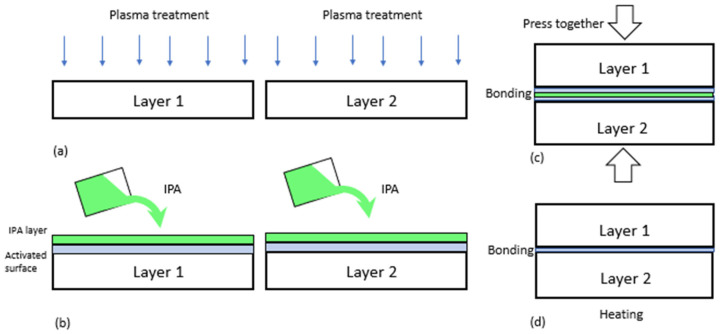
The existing approach of oxygen plasma bonding. (**a**) Surface activation process inside a vacuum chamber, (**b**) wetting of layers using solvents (e.g., IPA, DI water) to inhibit hydrophobic recovery to facilitate the alignment of two pieces or layers of materials, (**c**) bonding by pressing the two layers, (**d**) post heating to remove volatile components.

IPA is a commonly used solvent for cleaning and rinsing in the microfabrication process. In [76], super hydrophilicity can be achieved by soaking PDMS in isopropanol (IPA) (6, 12, and 24 h, respectively), followed by the plasma treatment with and without additives. IPA was also used as an additional solvent to produce a fine-pored PDMS, according to [72]. This is achieved at the PDMS preparation phase, specifically by mixing PDMS with IPA and water. Figure 1 summarizes the current procedure for the bonding of PDMS to PDMS (or other materials), in which IPA is taken as an example (some other solvents such as DI water [61] can replace IPA). The most important feature of this procedure is that the chemicals are coated on the plasma-treated surface (or activated surface) of PDMS, followed by the assembly of the two treated PDMS pieces and the heating of the assembly to evaporate the chemicals.

## 3. Our Approach

**Figure 2:**
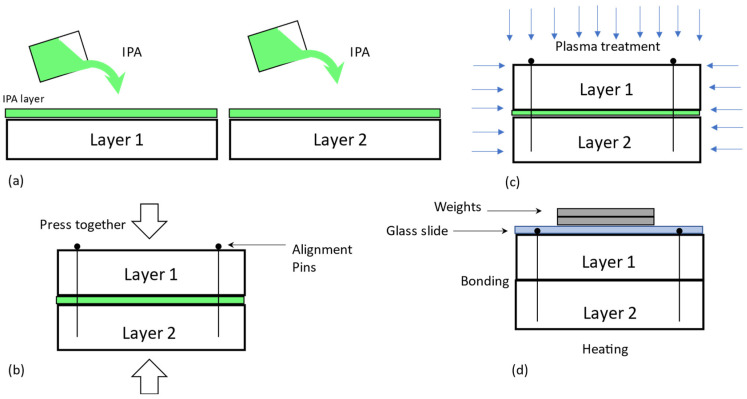
The proposed approach and bonding procedure. (**a**) Wetting of layers using IPA, (**b**) alignment process with the aid of alignment pins, (**c**) sandwiched layers placed inside the vacuum chamber for plasma treatment, (**d**) post heating to remove volatile components.

In our study, a new procedure for the bonding of PDMS to PDMS was developed, as shown in Figure 2. In this procedure, the IPA is coated on the surface of PDMS prior to the plasma treatment of the surface of PDMS (Figure 2a). After that, the two IPA-coated PDMS pieces are assembled (Figure 2b) to satisfy a particular alignment requirement (e.g., several microns). Alignment pins (specifically, Staples Round Push Pins Model#10559) were carefully inserted into four corners of the mold, and this process was performed under the microscope. A simple hand pressing was the only pressure applied during this process. The alignment pins were expected to produce an accurate alignment, to hold the layers together after pressing (avoiding slipping due to the presence of the solvent), and to reduce the shift between layers in the vacuum inside the plasma chamber. It is noted that an extreme level of shift can happen due to the presence of the solvent in vacuum in plasma treatment without alignment pins. Plasma treatment was then performed on the assembly (Figure 2c) at 50 W, 45 milli Torr oxygen for 30 s (Reactive Ion Etcher System, Torr International Inc). Subsequently, a glass slide with calibration weights (100–250 gm) was placed on top of the layers to provide sufficient pressure, giving a post-exposure bake (Figure 2d) of 30 min at 85 °C (Binder FED 115 large heating/drying oven). The bonded structure was placed at room temperature for 24 h before testing. This way of plasma-treating the contact surface of PDMS in the assembly works because PDMS is highly gas permissible [77,78,79,80,81] and IPA is a permeable liquid as well. In addition, PDMS also absorbs molecules from the sample liquids [82,83,84].

PDMS (Dow Corning Sylgard 184 Silicone Elastomer) was prepared by mixing both the hardener and elastomers in a 1:10 ratio using an electric stirrer (J-KEM Scientific, Inc, St. Louis, MO, USA) for 15 min and placed inside the vacuum chamber (Binder VD23 vacuum drying oven) for 1 h to remove all the air bubbles. The liquid PDMS was then poured into a glass plate and cured. It is noted that the double salinization process (the regular salinization protocol [85] was repeated twice in an interval of 2 h) was done inside the glass dish prior to the PDMS molding. We conducted these experiments numerous times, varying different parameters with each. Few samples were prepared for the final testing, and only 4 samples underwent testing from each group. A set of samples were cured (in this paper, cured or curing refers to the solidification process of a liquid or melt) with two methods in order to understand their effects on bonding strength: (1) at room temperature and atmospheric pressure for a minimum of 3 days called “Natural”, and (2) using the conventional oven bake (Binder FED 115 large heating/drying oven) at 85 °C for 6 h called “Oven”. After curing, PDMS samples (1000 mm thickness) were cut into 5 mm × 5 mm pieces for subsequent steps of bonding and assembly.

Both groups of samples from Natural and oven were then treated with IPA (70%) for bonding, as illustrated in Figure 2. Both groups of samples were also examined for the situation without IPA (i.e., with the bonding process of Figure 1) and with IPA (i.e., with the bonding process of Figure 2). To all the samples, a post-exposure bake at 85 °C was performed for 30 min, and after that, the samples were gradually cooled down to room temperature.

**Figure 3:**
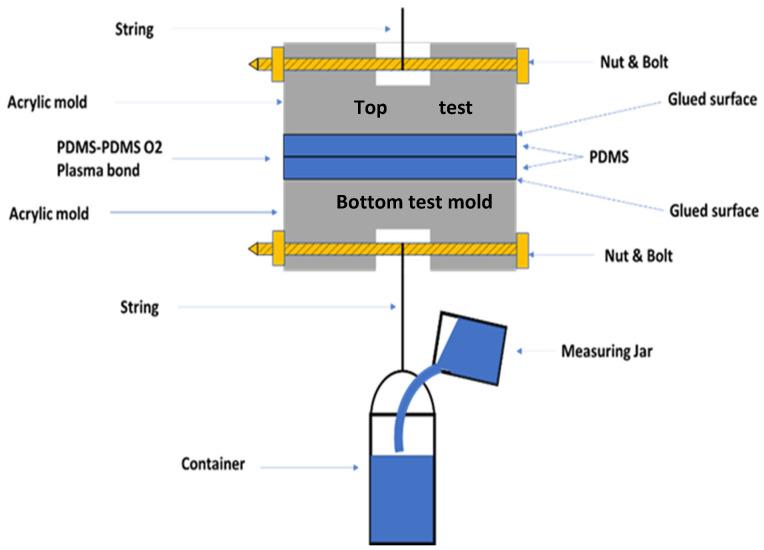
Tensile testing for measuring the bonding strength of the PDMS-to-PDMS.

The bonding strength of PDMS to PDMS was measured by using a custom-made simple setup for tensile testing (Figure 3). The bonded PDMS-to-PDMS slabs were glued (J-B Weld 50139 Adhesive) to the top test and bottom test molds, respectively, as illustrated in Figure 3. The mold is made of acrylic, which was chosen due to its availability. Each contact area between PDMS and the mold was 25 mm^2^. Bolts were inserted inside the mold, as illustrated in Figure 3. The whole test system was cured in a vacuum chamber for 24 h. After that, the bonding strength was evaluated in the following way. The container connected to the sample was slowly filled with water until the release of the bond. The tensile force on the bond was the weight of the container’s water at the bond’s breakage point. The tensile force on the bond was the weight of the water in the container at the breakage point of the string. The tensile strength of the bond was then calculated by dividing the force by the contact area (i.e., 25 mm^2^). The foregoing measurement was performed at Canadian Light Source (CLS) facilities located in Saskatoon, SK, Canada.

## 4. Results and Discussions

Table 2 provides a summary of the experiment results, including the specific bond strengths of the samples. It can be seen in Table 2 that the (1) bond with the IPA treatment along with the curing in the oven is the strongest, (2) the bond with the IPA treatment is stronger than the bond without IPA regardless of the oven curing or natural curing, and (3) the oven curing nor the natural curing do not significantly affect the bonding strength without the IPA treatment. It can be seen in Table 2 that the highest bonding strength is about 3 MPa. Glue failure occurred (2 times), which may have been due to the compatibility issue. There may be a significant difference in adhesion between the glue and the PDMS. Another cause is perhaps related to the difference in elasticity between the PDMS and the glue, which further leads to their different stretching ratios.

Elsewhere [86], we built a prototype of the so-called membrane switch valve made from PDMS, to which the bonding process includes IPA treatment. The PDMS membrane with microchannels was first bonded to a microscopic glass slide. A leakage test was performed using a standard syringe pump and deionized water, and no leakage was found. To further evaluate the plasma treatment (i.e., the presence of plasma), we experimented with a similar protocol as illustrated in Figure 2, only with a vacuum and IPA but no presence of plasma. The result obtained has not produced any bond characteristics (bonding strength and leakage) comparable with the bond characteristics in the bonding process involving the IPA treatment and plasma. This suggests that the presence of plasma is a necessary condition to increase the bonding strength. Another experiment was conducted by replacing IPA with ethanol and by following the procedure in Figure 2, the results of which show no meaningful improvement in the bonding strength.

**Figure 4:**
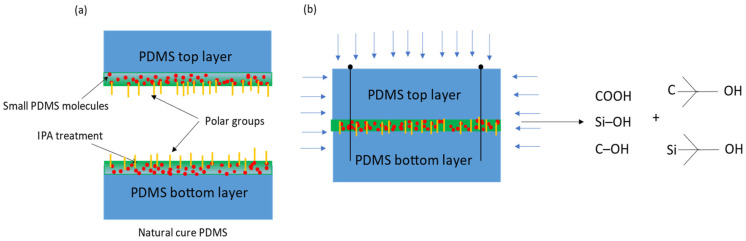
The surface treatment of PDMS using IPA, (**a**) showing small molecules along with polar groups after IPA treatment, (**b**) showing bonding formation.

The mechanism for the IPA treatment along with the proposed bonding procedure in Figure 2 is speculated. The surface of PDMS is dissolved by IPA, leading to the liberation of small PDMS molecules, these small molecules are then able to diffuse and cross-link with one another, as shown in Figure 4. According to the literature [87], Poly(vinyl alcohol) (PVA) can effectively adsorb on the surface of PDMS, subsequently producing γ-OH after plasma treatment. Due to the similar structure of PVA and IPA, it is possible that IPA will also produce γ-OH, Si-C(CH_3_)_2_-OH and C-C(CH_3_)_2_-OH. Further, due to the increase in the hydroxyl group (OH), the hydrogen bond is increased, resulting in an increase in the binding force. The post-heating process in the vacuum chamber further aids the diffusion and cross-linking processes, as well as evaporating the IPA and solidifying the bonds between PDMS molecules. PDMS with a smaller molecular weight is more soluble and therefore promotes successful bonding with functional groups, particularly hydroxyl groups. Conversely, PDMS with a larger molecular weight has a larger molecular size, resulting in fewer docking ports available for other molecules to be grafted, which can weaken the bond formed after IPA wetting. However, it is worth noting that such strengthening is presumed to be limited to the PDMS-to-PDMS interface, so the preference is given to PDMS with a lower molecular weight. On the other hand, a larger molecular weight has been shown to contribute to stronger bonding between PDMS and silicon glass plates, due to a greater abundance of interfacial interactions and longer free chains, as demonstrated in the studies and reported in [86,88].

## 5. Conclusions

In this paper, we proposed a novel bonding approach for PDMS–PDMS using surface modification with IPA solution. It is noted in the literature that the IPA solution has been used for cleaning purposes but not for PDMS bonding. In our approach, the treated samples were first aligned and pressed together into an assembly; then, the assembly underwent plasma treatment, which took place in a vacuum chamber; finally, the plasma-treated assembly is heat treated. The experiment has shown the effectiveness of the proposed approach in terms of improving both bonding strength and sealing. Specifically, the bonding strength can achieve 3 MPa between two naturally cured PDMS pieces. The sealing strength, though not quantitatively measured, is acceptable to our work to build the so-called integrated microfluidic circuit (IMC) [89], and it is perhaps acceptable to most of the microfluidic applications according to Table 1, where a comparison of the bonding strengths using different methods in literature is given.

To the best of our knowledge, our process is also unique in terms of the order of steps. First, two surface-treated PDMS pieces are assembled, followed by the plasma treatment of the assembly. The generic process (Figure 1) with many other approaches is in reverse order. In fact, this order is significant to the bonding of PDMS–PDMS. In our observation, there is an inherent problem with the generic process; that is, during the alignment process, the modified surface property may be recovered to its original status as well as geometrically distorted. Furthermore, the bonding characteristics highly depend on (1) the preparation of the PDMS (how it is cured), (2) pre-wetting with IPA or other solvents, (3) plasma treatment, and (4) post-heat treatment. The main advantages of our approach are the efficient alignment of multi-layered micro-fabricated structures, highly irreversible bonding strength, and less lead time in the micro-fabrication process. However, there are still some issues with our approach that need to be addressed in the future. One issue is that the top layer tends to deform during the plasma-treatment process, which makes it difficult to bond for high aspect ratio structures, and which may contribute to glue failure. Another issue is that there are some difficulties (e.g., a longer time) when creating a vacuum inside the plasma chamber because IPA is a liquid.

There are several future studies related to our approach. First, there is a need to understand how plasma activation can affect the (internal) contact surface of two PDMS pieces from their assembly. It may be helpful to improve this understanding by using the effect of plasma treatment on liquids (especially IPA). Plasma treatment under atmospheric conditions (non-vacuum) needs to be carried out to investigate the role of vacuums towards IPA to create effective bonding characteristics. This could also provide a clear understanding of the quantity of IPA that affects the bonding strength. Additionally, PDMS permeability under vacuum and plasma with respect to IPA needs further evaluation. Second, the measurement of temperature change inside the vacuum chamber during plasma treatment may provide further information to improve our approach. Third, the effect of the internal contact surface area and layer thickness of the PDMS on bonding strength needs to be studied, which helps to improve our approach further. Fourth, a comprehensive leakage test needs to be carried out with quantitative analysis to understand the scope of applications of the PDMS microfluidic devices. Fifth, the bonding strength using different types of epoxy glues [90,91] and UV-curable adhesives (NOA74) [92] needs to be evaluated. Sixth, a relationship in terms of bonding strength with solvents other than IPA (ethanol, methanol, acetone, chloroform etc.) needs further analysis to achieve numerical data. Detailed structure analysis of the w.r.t swelling ratio before and after plasma treatment need to be imaged and evaluated. Finally, shear strength and peel tests need to be taken for a more complete evaluation of the overall bond quality of the surface in contact.

## Figures and Tables

**Table 1:** Comparison of the bonding strengths using various methods.

Method	Substrate	Maximum Bond Strength	Ref.
Oxygen Plasma	PDMS–PDMS	0.510 MPa	[57]
Nitrogen Plasma	PDMS-SU8	0.428 MPa	[58]
Corona Discharge	PDMS–PDMS	0.290 MPa	[59]
Partial Curing of PDMS	PDMS–PDMS	0.651 MPa	[59]
Vacuum Airbag Lamination (VAL)	PDMS-Glass	0.739 MPa	[60]
Plasma Enhanced	PDMS-PARYLENE	1.4 MPa	[61]
Argon Plasma	PDMS–PDMS	1.9 MPa	[62]

**Table 2:** Bonding strengths of the samples in the experiment.

	Type of Treatment	Type of Curing	Load (MPa)	Failure Mode
Sample 1	With IPA	Natural	2.821	PDMS–PDMS
		Oven	1.373	PDMS–PDMS
	Without IPA	Natural	0.392	PDMS–PDMS
		Oven	0.235	PDMS–PDMS
Sample 2	With IPA	Natural	2.668	Glue Failure
		Oven	0.686	PDMS–PDMS
	Without IPA	Natural	0.372	PDMS–PDMS
		Oven	0.247	PDMS–PDMS
Sample 3	With IPA	Natural	2.786	Glue Failure
		Oven	0.941	PDMS–PDMS
	Without IPA	Natural	0.239	PDMS–PDMS
		Oven	0.215	PDMS–PDMS
Sample 4	With IPA	Natural	3.060	PDMS–PDMS
		Oven	1.020	PDMS–PDMS
	Without IPA	Natural	0.353	PDMS–PDMS
		Oven	0.400	PDMS–PDMS

## Data Availability

Data will be made available based on request.
